# An Energy-Efficient UWB Transmitter with Wireless Injection Locking for RF Energy-Harvesting Sensors

**DOI:** 10.3390/s21041426

**Published:** 2021-02-18

**Authors:** Jun-Tae Kim, Bo-Ram Heo, Ickjin Kwon

**Affiliations:** Department of Electrical and Computer Engineering, College of Information Technology, Ajou University, Suwon 16499, Korea; sso00072@ajou.ac.kr (J.-T.K.); ramob94@ajou.ac.kr (B.-R.H.)

**Keywords:** RF energy harvesting, ultrawideband, transmitter, RF clock generator, pulse generator, energy-harvesting sensor

## Abstract

An ultralow-power ultrawideband (UWB) transmitter with an energy-efficient injection-locked radio frequency (RF) clock harvester that generates a carrier from an RF signal is proposed for RF energy-harvesting Internet-of-Things (IoT) sensor applications. The energy-efficient RF clock harvester based on the injection-locked ring oscillator (ILRO) is proposed to achieve optimal locking range and minimum input sensitivity to obtain an injection-locked 450 MHz clock in ultralow-power operation. A current-starved inverter-based delay stage is adopted that allows delay adjustment by bias voltage to minimize dynamic current consumption while maintaining a constant delay regardless of changes in process, supply voltage, and temperature (PVT). To minimize static current consumption, a UWB transmitter based on a digital-based UWB pulse generator and a pulse-driven switching drive amplifier is proposed. The proposed injection-locked RF clock harvester achieves the best RF input sensitivity of −34 dBm at a power consumption of 2.03 μW, enabling energy-efficient clock harvesting from low RF input power. In ultralow-power operation, a 23.8% locking range is achieved at the RF injection power of −15 dBm to cope with frequency changes due to PVT variations. The proposed UWB transmitter with RF clock harvester achieves the lowest energy consumption per pulse with an average power consumption of 97.03 μW and an energy consumption of 19.41 pJ/pulse, enabling operation with the energy available in RF energy-harvesting applications.

## 1. Introduction

Energy-harvesting technology that derives energy from the surrounding environment, such as from solar radiation [1], pressure [2,3], friction [4], radio frequency (RF) energy [5,6], vibration [1,7], temperature [1,8], and light [9], is one of the core technologies of Internet-of-Things (IoT) sensors that operate without the need for power or batteries [10,11,12,13]. Since the power available for energy harvesting is quite limited, power consumption of less than a few microwatts is required in energy-harvesting circuits [14,15]. However, in order to generate the reference clock required for the transmitter, a clock generator consisting of a power-consuming oscillator and a phase-locked loop (PLL) is required [16]. Especially in the wireless interface, it is necessary to reduce the clock generation block, because it consumes a significant portion of the power consumption [17]. Instead of the local oscillator based on the conventional PLL, which consumes a lot of power, extracting the clock from the RF carrier can generate an energy-efficient frequency clock with high accuracy [18,19,20]. However, in order to generate the clock using the existing injection-locked frequency divider, the minimum injection power to obtain the injection-locked output signal requires relatively higher power than the input power obtained from ambient RF energy harvesting [21,22,23]. In addition, the power consumption of the injection-locked frequency divider increases in order to obtain a wide locking range to cope with process, supply voltage, and temperature (PVT) variations. The ultrawideband (UWB) transmitter based on a low-power pulse generator is suitable for RF energy-harvesting sensor applications because it enables low-power wireless transmission. However, the drive amplifier that transmits the output to the antenna needs high power consumption, and the linear drive amplifier consumes static current during the standby operation, which is the main cause of low efficiency [24,25,26,27,28,29,30].

In this paper, we propose an ultralow-power UWB transmitter design based on an energy-efficient injection-locked RF clock harvester for IoT sensors driven by RF energy harvesting. In order to achieve the minimum input sensitivity and maximum locking range for the injection-locked RF clock in ultralow-power operation, an injection-locked RF clock harvester based on the injection-locked ring oscillator (ILRO) is proposed. The proposed injection-locked RF clock harvester achieves the best RF input sensitivity of −34 dBm at a power consumption of 2.03 μW, enabling energy-efficient clock harvesting from low RF input power. In ultralow-power operation, a 23.8% locking range can be obtained at the RF injection power of −15 dBm to cope with frequency changes due to PVT variations. The proposed UWB transmitter with RF clock harvester achieves the lowest energy consumption per pulse with an average power consumption of 97.03 μW and an energy consumption of 19.41 pJ/pulse, enabling operation with the energy available in RF energy-harvesting applications.

## 2. Proposed RF Clock Harvester Design

**Figure 1:**
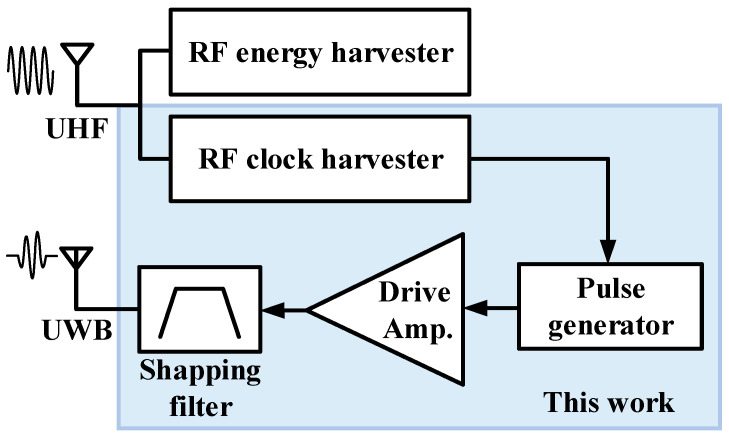
Block diagram of the proposed ultrawideband (UWB) transmitter with the injection-locked radio frequency (RF) clock harvester.

Figure 1 shows a block diagram of the proposed UWB transmitter with injection-locked RF clock harvester. It consists of an RF clock harvester, a pulse generator, and a drive amplifier. The energy-efficient RF clock harvester is based on injection-locked ring oscillator (ILRO). A digital UWB pulse generator and a pulse-driven switching drive amplifier are adopted to eliminate the static current in standby mode.

The RF energy harvester receives UHF RF signals from a dedicated RF source such as an RFID reader or collects RF energy from ambient RF sources such as cellular base stations. The power available from a dedicated RF source decreases considerably as the distance between the sensor and the dedicated source increases. The average RF power available in the RF cellular (LTE, GSM) and ISM bands has a wide range from −25 to 0 dBm [15]. Therefore, the proposed RF clock harvester is designed to operate at an input power of −25 dBm, which is the minimum average power that can be obtained from available ambient RF energy sources. A 450 MHz clock is generated from a 900 MHz RF signal by the divide-by-2 operation of the ILRO-based RF clock harvester. Since multiple input injection [31,32,33,34,35,36,37] requires additional phase control circuits and consumes a lot of power, single input injection with low power consumption is adopted for injection-locked RF clock harvester. In order to achieve a relatively wide locking range in low-power operation and to achieve a small chip area, a ring oscillator is adopted instead of an inductor-capacitor (LC) oscillator [38,39].

**Figure 2:**
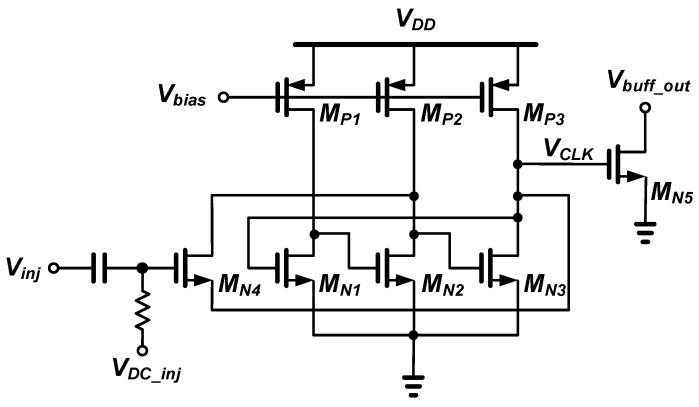
Schematic of the injection-locked RF clock harvester.

Figure 2 shows a schematic of the injection-locked RF clock harvester with ILRO-based divide-by-2 frequency divider. Each single-ended delay stage consists of an n-channel metal-oxide-semiconductor (NMOS) inverter and a p-channel metal-oxide-semiconductor (PMOS) current source, and the current is controlled by the gate bias voltage *V_bias_*. The ring oscillator consists of three stages, and the RF injection signal is input to the gate of the injection transistor *M_N4_* located between the second and third stages. An open-drain NMOS common-source stage is used as the output buffer for the measurement.

The locking range is proportional to the width of the injection transistor *M_N4_* and inversely proportional to the total capacitance of the injection node [40].

(1) $$\mathrm{locking}\;{\mathrm{range}}_{\max}\;\approx \;\frac{\alpha_{inj}V_{inj}}{C_{inj}}$$

where *α_inj_* is an injection factor proportional to the width and gate bias voltage of the injection transistor *M_N4_*. *V_inj_* is the magnitude of the RF injection voltage and *C_inj_* is the total capacitance of the injection node. In order to enable locking operation even at low RF injection power, it is necessary to increase *α_inj_* without increasing *V_inj_*.

As the width of the injection transistor *M_N4_* increases, *α_inj_* increases, but the injection node capacitance *C_inj_* also increases. There is a design trade-off between the width of *M_N4_* and the locking range. Therefore, in order to obtain the maximum locking range, it is necessary to design the width of *M_N4_* as an optimal value. Moreover, as the gate bias voltage *V_DC_inj_* of the injection transistor increases, *α_inj_* increases and the locking range increases. However, the output resistance of the injection transistor decreases in inverse proportion to *V_DC_inj_*, and because the injection current applied to the ring oscillator decreases, the lock range decreases. There is also a design trade-off between the *V_DC_inj_* of the injection transistor and the locking range. Therefore, in order to obtain the maximum locking range, not only the width of the injection transistor *M_N4_* but also the gate bias voltage *V_DC_inj_* should be designed to be an optimum value.

**Figure 3:**
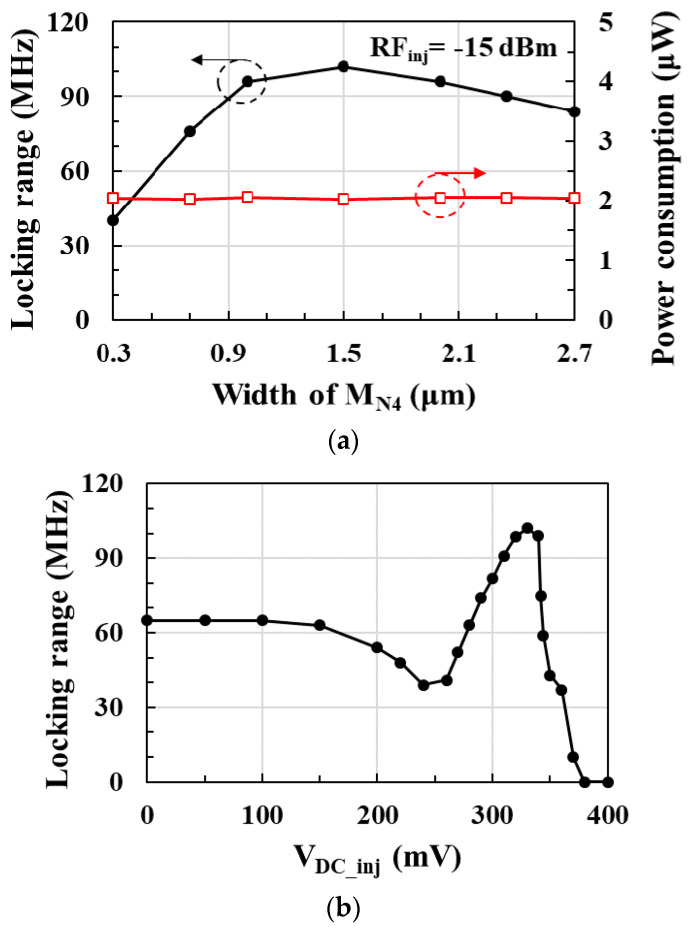
Locking range and power consumption of the RF clock harvester according to the (**a**) width and (**b**) gate bias voltage V_DC_inj_ of injection transistor M_N4_.

Figure 3a shows the postlayout-simulated locking range and power consumption of the RF clock harvester according to the width of the injection transistor *M_N4_* at the RF injection power of −15 dBm. As the width of *M_N4_* increases, there is a maximum value of the locking range due to the trade-off between the increase of the injection current and the increase of the capacitance. Since the current consumption does not change with the width of *M_N4_*, the locking range can be improved without increasing the current consumption. Therefore, optimizing the width of *M_N4_* is more effective to improve the locking range without increasing power consumption. In this design, the width of *M_N4_* is optimized to 1.5 μm, which improves the locking range from 40 to 102 MHz by 155% without increasing power consumption.

**Figure 4:**
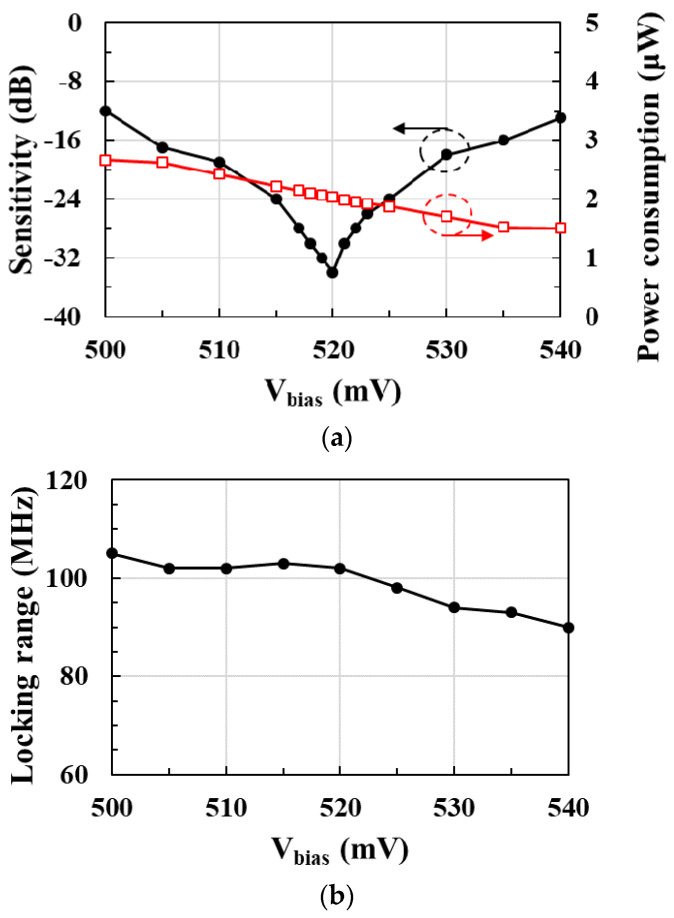
(**a**) Input sensitivity and power consumption; (**b**) locking range of the RF clock harvester according to the gate bias voltage *V_bias_* of the PMOS current source.

Figure 3b shows the postlayout-simulated locking range according to the gate bias voltage *V_DC_inj_* of the injection transistor *M_N4_* at the RF injection power of −15 dBm. Since *α_inj_* increases with the gate bias voltage of the injection transistor, and the output resistance of the injection transistor decreases in inverse proportion to the gate bias voltage, there is an optimal value of *V_DC_inj_* to achieve the maximum locking range as shown in Figure 4. At gate bias voltages greater than 380 mV, oscillation does not occur due to the decrease in the output resistance of the injection transistor, and the 450 MHz injection lock output signal cannot be obtained. In this design, the gate bias voltage *V_DC_inj_* of the injection transistor is optimized to 330 mV to achieve a maximum locking range of 102 MHz.

As the width of the PMOS current source increases, the locking range improves, but the current consumption of the ring oscillator increases in proportion to the width [41]. Moreover, as the width of PMOS increases, the locking range does not increase any more due to the increase in injection node capacitance *C_inj_*. In this design, for optimal trade-off design between current consumption and locking range, the width of the PMOS current source is designed to be 0.26 μm, and the current is adjusted by controlling the gate bias voltage.

When the gate bias voltage *V_bias_* of the PMOS current source is lowered, the current in each inverter stage of the ring oscillator increases and the resistance of the PMOS active load decreases, so the gate delay of the ring oscillator decreases and the free-running frequency increases. In order to minimize the input sensitivity—which is the minimum RF injection power for the injection-locked 450 MHz output—it is necessary to design the free-running frequency to be 450 MHz. Therefore, in order to obtain minimum RF input sensitivity, *V_bias_* is designed as an optimum value so that the free-running frequency is tuned to 450 MHz.

Figure 4a shows the postlayout-simulated sensitivity and power consumption of the RF clock harvester according to the gate bias voltage *V_bias_* of the PMOS current source. Input sensitivity, which is the minimum RF injection power to obtain an injection-locked 450 MHz clock, is obtained with a minimum of −34 dBm at *V_bias_* of 520 mV.

Figure 4b shows the postlayout-simulated locking range of the RF clock harvester according to *V_bias_* at the RF injection power of −15 dBm. At *V_bias_* below 520 mV, current consumption increases, but the lock range remains almost constant. However, at *V_bias_* over 520 mV, the current consumption decreases, but the locking range also decreases. Therefore, the optimum locking range is achieved at *V_bias_* of 520 mV, which can obtain minimum input sensitivity.

## 3. Proposed UWB Transmitter Design

### 3.1. UWB Pulse Generator

For energy-harvesting applications, it is necessary to minimize the current dissipated by the UWB pulse generator at low supply voltage while maintaining a constant UWB bandwidth for ultralow-power operation. In the proposed UWB pulse generator, a digital logic-based pulse generator operating at a supply voltage as low as 1 V is adopted to eliminate static currents in standby mode. A delay stage based on current adjustable current mirror and current starved inverter is adopted to minimize the consumption of dynamic current and maintain a constant delay regardless of changes in process, supply voltage, and temperature in the delay stage.

**Figure 5:**
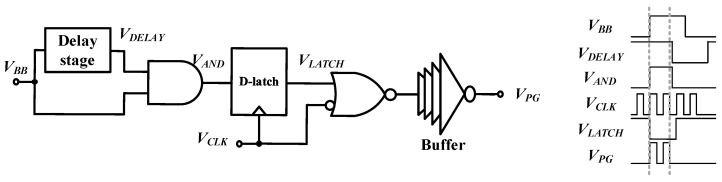
Block diagram and timing diagram of the pulse generator.

Figure 5 shows the block diagram and timing diagram of the pulse generator. The proposed pulse generator is based on a digital logic circuit, and the bandwidth of the pulse is controlled by an adjustable delay stage circuit. The baseband signal *V_BB_* is generated by the sensor interface for sensing information that appears as a change in sensor resistance or capacitance. The timing diagram of each block describes the operation of the UWB pulse generator. The baseband signal (*V_BB_*) applied to the pulse generator is delayed by a constant time of about 5 ns to secure a bandwidth of 400 MHz or more in the delay stage. The delayed signal (*V_DELAY_*) and the original signal are input to the complementary metal-oxide-semiconductor (CMOS) AND gate to output the window (*V_AND_*) signal. This signal is synchronized with the output signal of a clock generator (*V_CLK_*) to output *V_LATCH_* having a window corresponding to two cycles of *V_CLK_*. *V_LATCH_* and the inverted *V_CLK_* are input to the CMOS NOR gate and output two pulses (*V_PG_*) synchronized to the 450 MHz clock.

**Figure 6:**
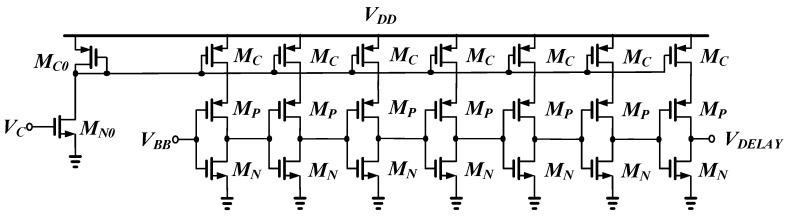
Schematic of the delay stage.

Figure 6 shows the schematic of the delay stage of the UWB pulse generator. The delay stage consists of a current adjustable current mirror and a seven-stage current starved inverter (CSI). The CSI stage consists of a current source and an inverter, and the maximum dynamic current of the inverter is determined by the current source [42]. The current source of each CSI stage of the delay stage is determined by the current mirror, and this current is controlled by the control voltage (*V_C_*) of the reference current source. The maximum delay time of the delay stage is designed to be less than 5 ns to operate as a UWB-based pulse generator with a large bandwidth above 400 MHz.

**Figure 7:**
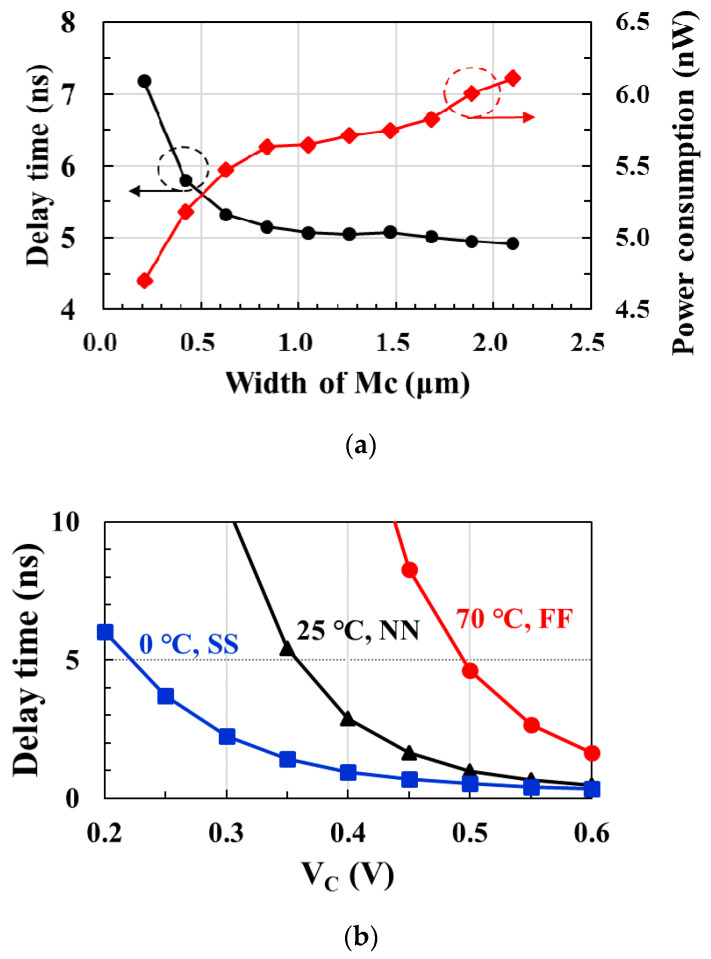
(**a**) Delay time and power consumption of the delay stage according to the width of *Mc*; (**b**) delay time with control voltage *V_C_* at different corner models and temperature conditions.

Figure 7a shows the postlayout-simulated delay time and power consumption according to the width of *M_C_* in the delay stage. When the width of the *M_C_* increases in the range of 1.0 μm or more, the power consumption increases, but the delay remains almost constant at 5 ns. Therefore, the size of the *M_C_* is optimized to 1.05 μm to maintain a 5 ns delay while consuming less power.

In low-power designs, there is a significant problem with delay changes due to process, voltage, and temperature (PVT) variations. Figure 7b shows the postlayout-simulated delay time according to the *V_C_* of the delay stage at different corner models and temperature combinations. Even if the delay time changes owing to PVT variations, the delay time of 5 ns can be kept constant by adjusting *V_C_* from 0.22 to 0.5 V.

### 3.2. Drive Amplifier

Drive amplifiers, which consume most of the current in the transmitter, consume significant amounts of current owing to static currents even when there is no input signal [16,26,27,28,29,30]. Therefore, it is necessary to reduce the static current of the drive amplifier for the design of low-power sensor interfaces with duty-cycling operation. The proposed UWB transmitter adopts a switching amplifier driven by the output of a digital pulse generator to reduce static current.

**Figure 8:**
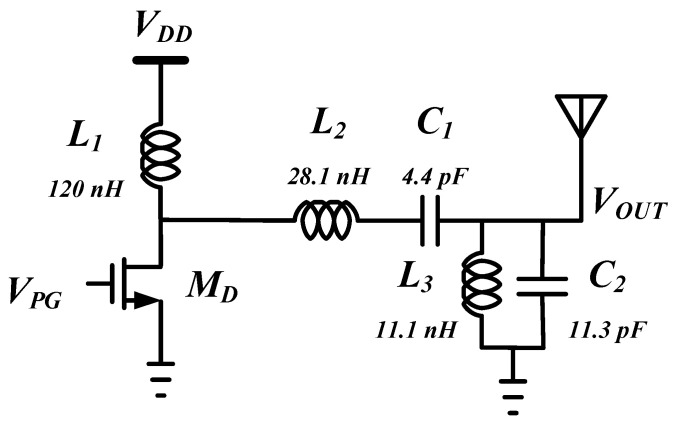
Schematic of the switching drive amplifier with off-chip LC pulse shaping filter.

Figure 8 shows the schematic of the driving amplifier. It is composed of switching transistor *M_D_*, load inductor *L_1_*, and second-order LC filter for pulse shaping. In standby mode, the *M_D_* is turned off so that static current does not flow. When the output of the pulse generator is high, the *M_D_* turns on, and the on-resistance decreases rapidly. A rectangular wave pulse is applied to the gate of *M_D_* by a pulse generator to increase power efficiency. The second-order LC band-pass filter is configured off-chip to satisfy the output spectrum mask. For *L_1_*, a 120 nH off-chip inductor is used. The component values of the off-chip second-order LC filter for pulse shaping are shown in Figure 10. An output impedance matching network is required to deliver output power to an antenna with 50 Ω impedance. In order to eliminate the off-chip matching network, the width of the *M_D_* is optimized so that the output impedance of the switching amplifier is 50 Ω while satisfying the output power required when the switching transistor *M_D_* is turned on.

**Figure 9:**
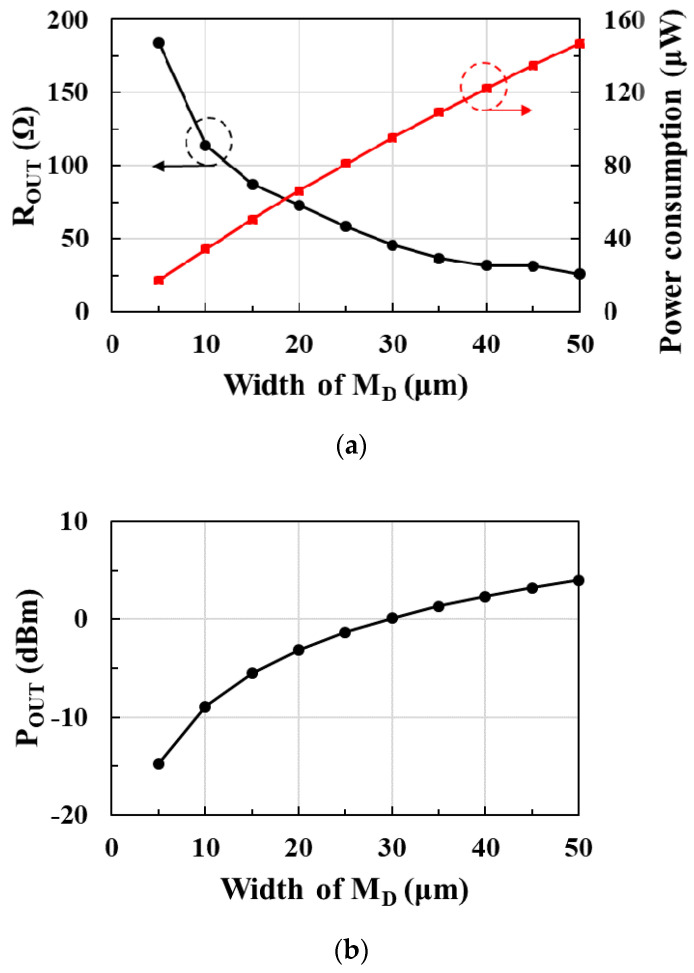
(**a**) Output resistance (R_OUT_), power consumption, and (**b**) output power (P_OUT_) of the drive amplifier according to the width of M_D_.

Figure 9a shows the postlayout-simulated output resistance *R_OUT_* and power consumption according to the width of the input transistor *M_D_* in the drive amplifier. *R_OUT_* is the real part of the drive amplifier’s output impedance *Z_OUT_*. As the width of *M_D_* increases, *R_OUT_* decreases but power consumption increases in proportion to the width. Figure 9b shows the postlayout-simulated output power *P_OUT_* of the drive amplifier according to the width of *M_D_*. As the width of *M_D_* increases, *P_OUT_* increases. Therefore, in order to satisfy the 0 dBm output power and obtain a 50 Ω output resistance, the width of the drive amplifier is designed to be 30 μm. A buffer is inserted between the pulse generator and the drive amplifier to drive the input transistors of the drive amplifier. The buffer consists of four inverters, and the size of each inverter is designed to maintain constant pulse width by optimizing rising and falling time.

## 4. Results and Discussion

**Figure 10:**
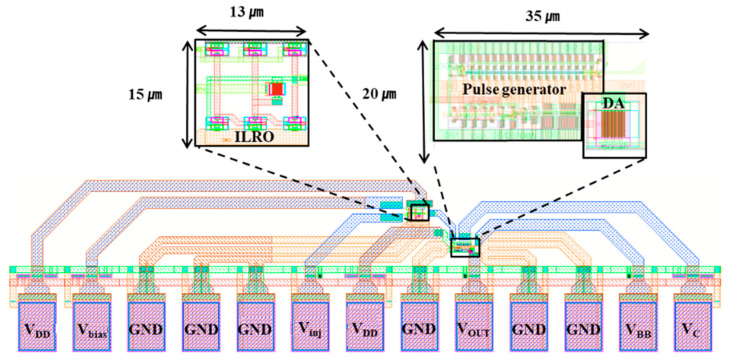
Chip layout of the proposed ultrawideband (UWB) transmitter with RF clock harvester.

The proposed UWB transmitter with injection-locked RF clock harvester was designed in 65 nm CMOS process. Figure 10 shows a chip layout of the proposed UWB transmitter with RF clock harvester. The area of the RF clock harvester is 13 × 15 μm^2^ and the area of the UWB transmitter consisting of a pulse generator and drive amplifier is 35 × 20 μm^2^. For energy-harvesting applications, the transmitter is designed to operate on low supply of 1 V.

**Figure 11:**
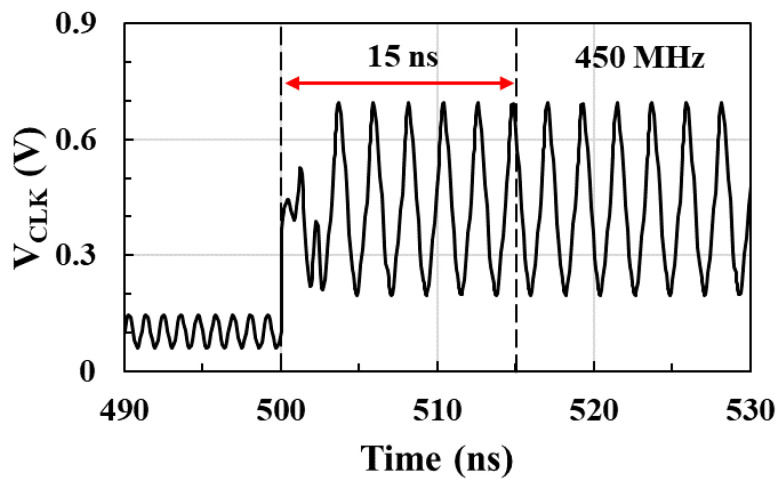
Output waveform of the RF injection-locked clock harvester and setup time when *V_DD_* is switched from 0 to 1 V.

Figure 11 shows the postlayout-simulated output waveform and setup time of the RF clock harvester when *V_DD_* is switched from 0 to 1 V. Output signal of the clock harvester is injection-locked by −15 dBm and 900 MHz RF input power. The setup time of the transmitter includes the start-up time and lock-in time of the clock harvester. The setup time is measured as the time taken to obtain a stable 450 MHz output waveform after *V_DD_* is applied, and the measured setup time is 15 ns.

**Figure 12:**
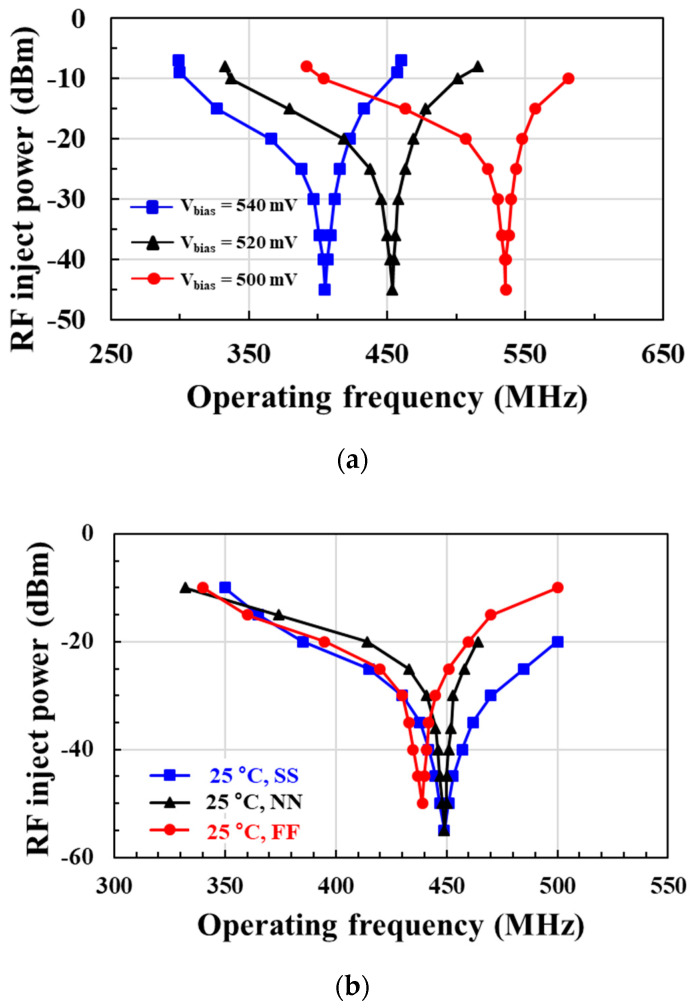
Locking range and sensitivity at (**a**) different *V_bias_* and (**b**) different corner models and temperature combinations.

Figure 12a shows the postlayout-simulated locking range and sensitivity of the RF clock harvester according to the operating output frequency. When the gate bias voltage *V_bias_* of the PMOS current source is lowered, the current of the ring oscillator increases, thus increasing the free-running frequency. Therefore, a lower *V_bias_* results in an injection-locked output at a higher frequency. The optimum *V_bias_* for obtaining the minimum input sensitivity differs according to the output frequency. The optimum *V_bias_* for the 450 MHz output frequency is 520 mV, achieving an input sensitivity of −34 dBm. A locking range of 23.8% is achieved at the RF injection power of −15 dBm.

Figure 12b shows the postlayout-simulated locking range and input sensitivity at different process corner and temperature combinations. In the RF clock harvester circuit, the *V_bias_* of the PMOS current source is adjusted according to the process corner and temperature combination for injection lock operation. The applied *V_bias_* was 520 mV for the 25 °C, normal/normal (NN) model; 435 mV for the 0 °C, slow/slow (SS) model; and 580 mV for the 70 °C, fast/fast (FF) model. The postlayout simulation result shows that an injection-locked 450 MHz output signal can be obtained for all process corner and temperature combinations at RF injection power below −25 dBm.

The proposed injection-locked RF clock harvester achieves −34 dBm RF input sensitivity at 2.03 μW power consumption, showing the best input sensitivity compared to the previous injection-locked frequency divider (ILFD). When −15 dBm RF power is injected, it achieves a locking range of 23.8%, allowing control of frequency changes due to PVT variations in ultralow-power operation.

**Figure 13:**
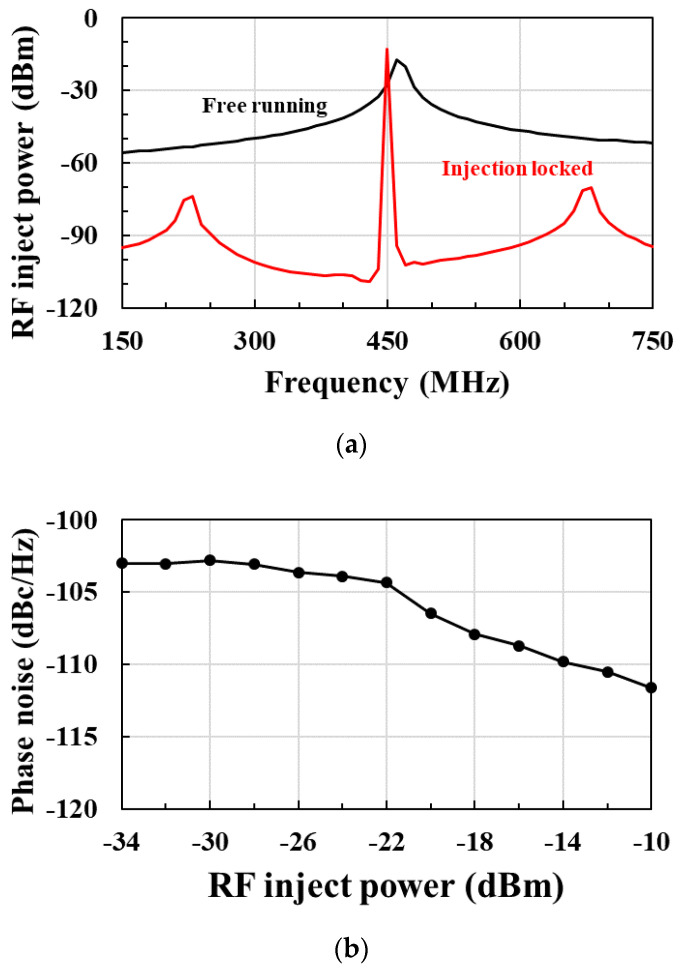
(**a**) Power spectral density (PSD) of the injection-locked RF clock harvester with and without RF injection power of −15 dBm; (**b**) phase noise of the RF clock harvester at 1 MHz offset according to the RF injection power.

Figure 13a shows the postlayout-simulated output power spectral density (PSD) of the RF clock harvester for free-running oscillation and injection-locked oscillation. The injection-locked oscillation output provides up to 40.2 dB reduction in phase noise compared to free-running oscillation. Figure 13b shows the postlayout-simulated phase noise of the RF clock harvester at 1 MHz offset according to the RF injection power. The phase noise is −108.24 dBc/Hz at RF injection power of −15 dBm. As the RF injection power increases, the phase noise is reduced.

**Figure 14:**
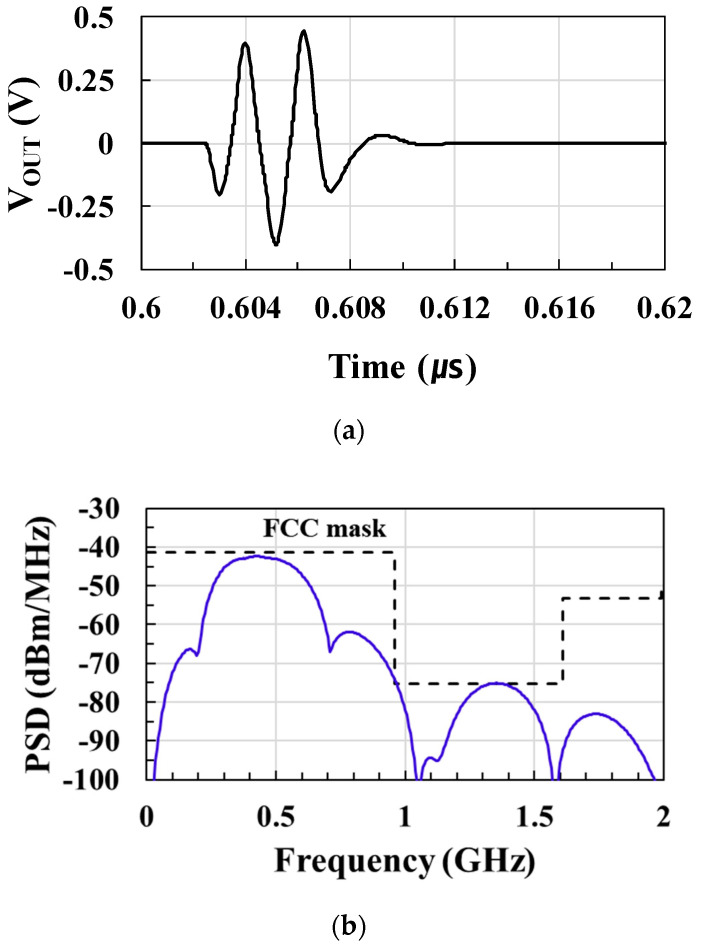
(**a**) Output pulse waveform of the UWB transmitter; (**b**) output power spectral density (PSD) of the UWB transmitter.

Figure 14a shows the postlayout-simulated output pulse waveform of the UWB transmitter with an output swing of 0.77 V_PP_. The baseband signal is applied to the pulse generator to output UWB pulses, and the output power and spectrum mask requirements are satisfied by the drive amplifier and second-order LC pulse shaping filter. Figure 14b shows the postlayout-simulated output power spectral density (PSD) of the UWB transmitter. The center frequency of the UWB pulse is 450 MHz and −10 dB bandwidth is 415 MHz. The output spectrum of UWB transmitters meets Federal Communications Commission (FCC) regulations.

Table 1 shows the performance summary of the proposed UWB transmitter and performance comparison with other works. The average total power consumption of the designed transmitter is 97.03 μW at 1 V supply voltage, and the energy consumption is 19.41 pJ/pulse. The proposed UWB transmitter with the RF clock harvester achieves the lowest energy consumption per pulse compared to previous UWB transmitters.

## 5. Conclusions

An ultralow-power UWB transmitter with an energy-efficient injection-locked RF clock harvester that generates a 450 MHz carrier from a 900 MHz RF signal has been proposed for RF energy-harvesting IoT sensor applications. The injection-locked RF clock harvester based on the ILRO proposed in this study achieves optimum locking range and minimum input sensitivity in ultralow-power operation. The UWB transmitter was designed based on a digital logic-based UWB pulse generator and a pulse-driven switching drive amplifier to achieve minimum energy consumption per pulse. The proposed UWB transmitter with injection-locked RF clock harvester was designed in 65 nm CMOS process. The proposed injection-locked RF clock harvester achieves an RF input sensitivity of −34 dBm and a lock range of 23.8% at −15 dBm input power with 2.03 μW power consumption. The proposed UWB transmitter with RF clock harvester achieves the lowest energy consumption per pulse with an average power consumption of 97.03 μW and an energy consumption of 19.41 pJ/pulse.

## Figures and Tables

**Table 1:** Performance summary and comparison.

Reference	[16]	[24]	[43]	[44]	This Work
CMOS process (nm)	180	130	180	65	65
Supply (V)	1	1.2	1.8	1	1
Sampling rate (MHz)	5	100	0.3	1	5
UWB band (MHz)	400 @f_C_ = 450	629.8 @f_C_ = 496	5000 @f_C_ = 5500	4000 @f_C_ = 8000	415 @f_C_ = 450
Output swing (V_PP_)	0.75	0.9	0.35	0.12	0.77
Power Consumption (μW)	175	2700	11.62	300	97.03
Energy/pulse (pJ/pulse)	35	27	38	300	19.41
